# Identification of biomarkers related to sepsis diagnosis based on bioinformatics and machine learning and experimental verification

**DOI:** 10.3389/fimmu.2023.1087691

**Published:** 2023-06-28

**Authors:** Qianfei Wang, Chenxi Wang, Weichao Zhang, Yulei Tao, Junli Guo, Yuan Liu, Zhiliang Liu, Dong Liu, Jianqiang Mei, Fenqiao Chen

**Affiliations:** ^1^Hebei University of Chinese Medicine, Shijiazhuang, China; ^2^The First Affiliated Hospital ,Hebei University of Chinese Medicine, Shijiazhuang, China

**Keywords:** sepsis, gene, pathways, RNA, immune infiltration

## Abstract

Sepsis is a systemic inflammatory response syndrome caused by bacteria and other pathogenic microorganisms. Every year, approximately 31.5 million patients are diagnosed with sepsis, and approximately 5.3 million patients succumb to the disease. In this study, we identified biomarkers for diagnosing sepsis analyzed the relationships between genes and Immune cells that were differentially expressed in specimens from patients with sepsis compared to normal controls. Finally, We verified its effectiveness through animal experiments. Specifically, we analyzed datasets from four microarrays(GSE11755、GSE12624、GSE28750、GSE48080) that included 106 blood specimens from patients with sepsis and 69 normal human blood samples. SVM-RFE analysis and LASSO regression model were carried out to screen possible markers. The composition of 22 immune cell components in patients with sepsis were also determined using CIBERSORT. The expression level of the biomarkers in Sepsis was examined by the use of qRT-PCR and Western Blot (WB). We identified 50 differentially expressed genes between the cohorts, including 2 significantly upregulated and 48 significantly downregulated genes, and KEGG pathway analysis identified Salmonella infection, human T cell leukemia virus 1 infection, Epstein−Barr virus infection, hepatitis B, lysosome and other pathways that were significantly enriched in blood from patients with sepsis. Ultimately, we identified COMMD9, CSF3R, and NUB1 as genes that could potentially be used as biomarkers to predict sepsis, which we confirmed by ROC analysis. Further, we identified a correlation between the expression of these three genes and immune infiltrate composition. Immune cell infiltration analysis revealed that COMMD9 was correlated with T cells regulatory (Tregs), T cells follicular helper, T cells CD8, et al. CSF3R was correlated with T cells regulatory (Tregs), T cells follicular helper, T cells CD8, et al. NUB1 was correlated with T cells regulatory (Tregs), T cells gamma delta, T cells follicular helper, et al. Taken together, our findings identify potential new diagnostic markers for sepsis that shed light on novel mechanisms of disease pathogenesis and, therefore, may offer opportunities for therapeutic intervention.

## 1 Introduction

Sepsis occurs when pathogenic microorganisms and their toxins invade the blood circulatory system (1, 2). The resulting immune system hyperactivation produces a variety of inflammatory cytokines and inflammatory mediators that cause systemic inflammatory response syndrome, and this can lead to multiple organ failure and shock (3). Worldwide, approximately 31.5 million people develop sepsis each year, and the disease is fatal for approximately 5.3 million of them (4). Unfortunately, the number of sepsis cases and related deaths continues to increase each year.

The pathogenesis of sepsis is complex, and it is generally believed to be related to dysregulation of the pro-inflammatory/anti-inflammatory responses, coagulation disorders, bacterial and endotoxin translocation, and gene polymorphisms. The uncontrolled inflammatory response plays a particularly important role in the rapid disease progression that is characteristic of sepsis. However, although this is well established and disease biomarkers such as PCT, white blood cell (WBC) count, and C-reactive protein (CRP) are used to inform on the disease state (5), the currently available approaches are often insufficient for clinicians to predict, monitor, and respond to changes in the condition of patients in a timely manner and with sufficient data to improve clinical outcomes. Therefore, the development of improved diagnostic biomarkers reflecting inflammation to improve clinical treatment of patients with sepsis is an urgent unmet medical need.

Several recent studies have identified specific genes that are involved in the pathogenesis and progression of sepsis (6–8). However, to date, the diagnostic value of many genes in sepsis has not been investigated. Currently, few studies have reported on target genes and immune cells in the blood or tissues of patients with sepsis. In this study, four microarrays(GSE11755, GSE12624, GSE28750, GSE48080) were merged into one comprehensive dataset to identify genes that are differentially expressed in patients with sepsis. We identified three genes, COMMD9, CSF3R, and NUB1, that were differentially expressed in sepsis compared to non-pathological specimens, and we correlated the differential expression of these genes with the immune cell composition of immune infiltrate. These findings identify novel genes that may be relevant for the pathogenesis of sepsis and that may be useful as diagnostic or predictive biomarkers for early disease detection.

## 2 Methods

### 2.1 Animals specimens

This study used nine healthy, six-week-old male Sprague Dawley (SD) rats with body weight 200 ± 20 g obtained from the Beijing Weitong Lihua Laboratory Animal Technology Co., Ltd. The animals were reared in three separate cages (3 per cage) in the animal husbandry center with standard sterile feed and drinking water supply ad libitim, natural light, at room temperature 22-26°C, in humidity 45-65% and with bedding changed twice weekly to ensure the rats lived in a well-regulated, quiet, and clean environment. After 7 days of adaptive rearing, the formal experiment was carried out. Eating, drinking, activity level, and defecation were monitored daily. After all rats were confirmed to be healthy, they were assigned to either the control group (n = 3) 、LPS sepsis model group (n = 3)、Cecal ligation perforation model (CLP) sepsis model group (n = 3) into three groups using a random number table. Rats were intragastrically administered 2 mL of saline once a day for 7 consecutive days. One hour after the last administration, the control group was given intraperitoneal injection of 5ml/kg saline, LPS group was given intraperitoneal injection of endotoxin (LPS 2 mg/ml) 10 mg/kg (9), CLP (10)group was given cecal ligation and puncture. After 24 hours, samples were collected and weighed. The ileum tissue and serum were taken and stored at -80°C. The study was approved by the Ethics Committee of Hebei University of Chinese Medicine (Number:DWL2019023).

### 2.2 Quantitative Real-Time PCR assay

Ileum tissue or serum was processed to extract total RNA. Next, 2 ug of total RNA was denatured to use as template RNA for qRT-PCR (BIO-RAD, model CFX96) in a mixture containing Oligo dT Primer and random primers at 70°C for 5 min and then immediately cooled on ice. The processed RNA was then added to the reverse transcription reaction solution containing reaction buffer, MgCl2, PCR nucleotide mix, ribonuclease inhibitor, reverse transcriptase and nuclease-free water to bring the volume of the reaction solution to 20 µl. The reaction was mixed slowly, briefly centrifuged, and placed in the PCR machine using the following cycle: 25°C for 5 min (annealing), 42°C for 60 min (extension), 70°C for 15 min (inactivation), for cycles. Then the reaction was cooled on ice and stored at -20° C for later use. The Cq values of each target gene and the internal reference gene β-actin were obtained. The Q value of each target gene/the Q value of the first sample, that is, RQ==2-ΔΔCq, represents the relative quantitative value of the expression of each target gene, and the RQ value was used for statistical analysis. The primers were as follows: COMMD9:forward (5′- CATCAGAGCATTTCGTGGCG -3′) and reverse (5′- AAGGGCTGAACTGGAGAAGC -3′); CSF3R: for-ward (5′- TGAGGGAAACAGAAAGGCCC -3′) and reverse (5′- AGACCTAGGGGTGTAGCCTG -3′); NUB1: for-ward (5′- GAATGAAAACAAACGGCGGC -3′) and reverse (5′- TCTGCGCCATCCTTGAAAGT -3′); and beta-actin forward (5′- GCAGGAGTACGATGAGTCCG -3′) and reverse (5′- ACGCAGCTCAGTAACAGTCC -3′).

### 2.3 Western blot

Western blot was used to detect the expression levels of COMMD9, CSF3R, and NUB1 proteins in rat ileum tissue. The ileum tissue was taken and placed in a centrifuge tube, and the protein in the ileum tissue was extracted using RIPA lysate. The supernatant was centrifuged and the protein concentration was measured using BCA method. We added the protein solution in a 4:1 ratio to 5×Loading buffer, denatured it in a boiling water bath for 25 minutes, performed 10% SDS-PAGE electrophoresis, and transferred the PVDF membrane for 30 minutes. The membrane was placed in a TBST incubation tank, shaken at room temperature with skim milk, and sealed for 2 hours. We added the diluted first antibody and incubated it at 4 °C on a shaker overnight. We used TBST to elute 3 times, each time for 5 minutes. The secondary antibody was diluted with TBST in a ratio of 1:5000, incubated at room temperature for 2 hours, and colored using ECL method. Finally, we used Image J software to analyze the grayscale values of the bands, and the relative expression level of the target protein=the grayscale value of the target protein/β- Action grayscale value.

### 2.4 Microarray data

Human microarrays were obtained from the NCBI Gene Expression Omnibus (GEO; https://www.ncbi.nlm.nih.gov/geo/). GSE11755 including 31 specimens from patients with sepsis and 10 healthy control specimens, was on the foundation of the GPL570[HG-U133_Plus_2] Affymetrix Human Genome U133 Plus 2.0 Array; GSE28750 including 21 specimens from patients with sepsis and 20 healthy controls, was on the foundation of the GPL570[HG-U133_Plus_2] Affymetrix Human Genome U133 Plus 2.0 Array; GSE48080 including 20 specimens from patients with sepsis and 3 healthy controls, was on the foundation of the GPL4133 Agilent-014850 Whole Human Genome Microarray 4x44K G4112F (Feature Number version); and GSE12624 including 34 specimens from patients with sepsis and 36 healthy controls, was on the foundation of the GPL4204 GE Healthcare/Amersham Biosciences CodeLink UniSet Human I Bioarray. We combined the four datasets using the SVA package functionality of the R program, removing batch effects.

### 2.5 DEGs and identification of differentially expressed genes

In this study, the limma package of R software was used to identify DEGs from the processed microarray data. The filter conditions were: |log2 Fold change (FC)| > 2, FDR < 0.05. Genes that met these criteria were identified as DEGs.

### 2.6 Functional enrichment analyses

Gene Ontology (GO) analysis and Kyoto Encyclopedia of Genes and Genomes (KEGG) analysis were completed based on the classification of high- and low-risk patients using the “cluster Profiler” R package of R software in this study, and p < 0.05 was considered statistically significant.

### 2.7 Candidate diagnosis marker selection

We applied two machine learning algorithms, LASSO and Support vector machine (SVM), to predict sepsis status. LASSO is a compressed estimate that retains the advantages of subset shrinkage. It is a biased estimate for processing data with complex collinearity. LASSO determines whether the discrimination between sepsis samples and normal samples is significant through the “glmnet” package in R software.

SVM is a class of generalized linear classifiers that perform binary classification on data in a supervised learning manner. The RFE algorithm was used to screen the optimal genes from the metadata cohort, and SVM-RFE was used to screen suitable features to identify strong gene sets.

### 2.8 CIBERSORT analysis

To determine the relationship between immune cell populations and sepsis, we determined the responses of 22 immune cell populations using the CIBERSORT method and assessed the association between these immune cells and the expression of key genes in normal samples and in samples from patients with sepsis.

### 2.9 Statistical analysis

Gene expression differences between specimens from patients with sepsis and normal specimens were compared using Student’s t-test. The ROC curve and AUC were calculated using the R package “proc” to test the classification effect of key genes in specimens from patients with sepsis and normal specimens. Statistical analysis was performed using R software (version 4.2.1) and GraphPad Prism (version 9.0.0) software, ∗ indicates p<0:05, ∗∗ indicates p<0:01.

## 3 Results

### 3.1 Determination of DEGs in sepsis

**Figure 1:**
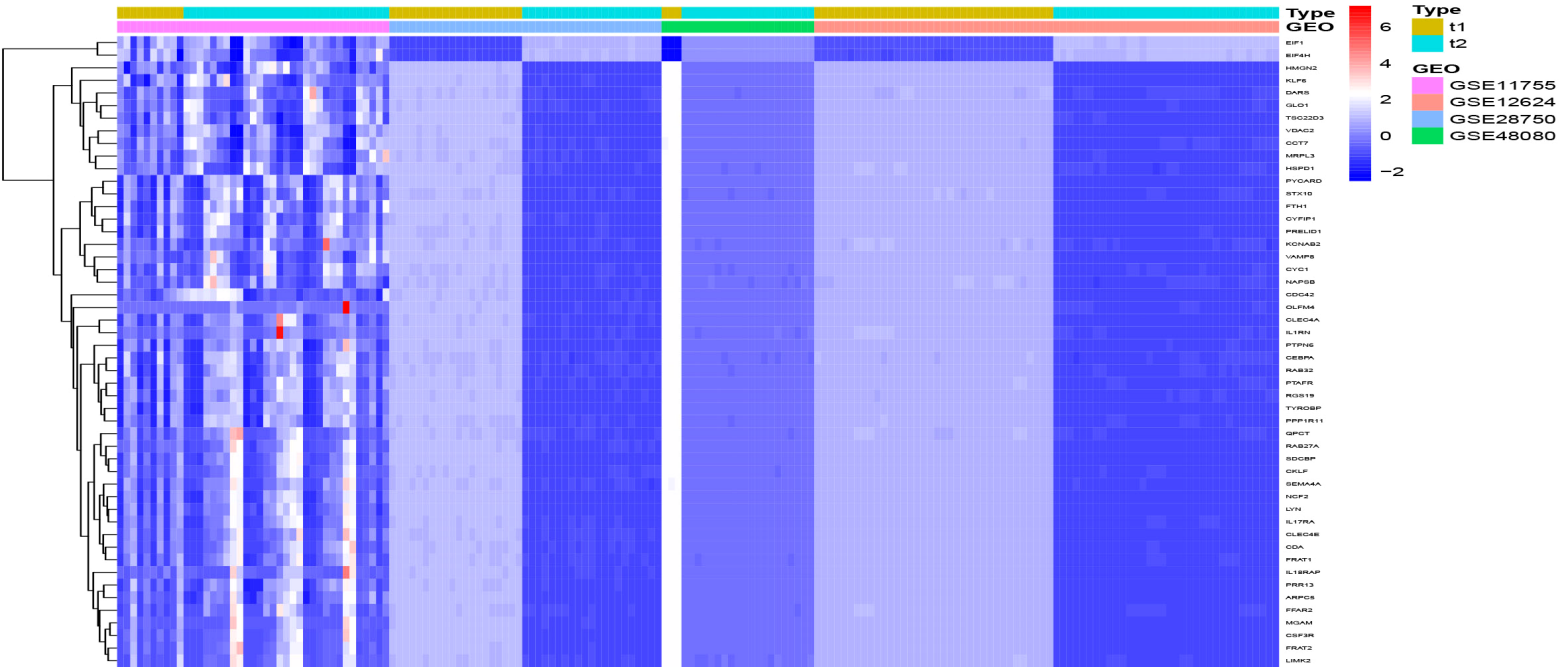
DEGs between Sepsis and control specimens, t1=normal, t2=sepsis.

In this study, four microarrays GSE11755, GSE12624, GSE28750, and GSE48080 were retrospectively analyzed that together included 106 specimens from patients with sepsis and 69 specimens from healthy subjects. After removing batch effects, the LIMMA software package was used to identify DEGS from metadata. A total of 50 DEGs were identified: 2 genes were significantly up-regulated and 48 genes were significantly down-regulated in samples from patients with sepsis compared to normal controls (**Figure 1**).

### 3.2 Functional enrichment analyses

**Figure 2:**
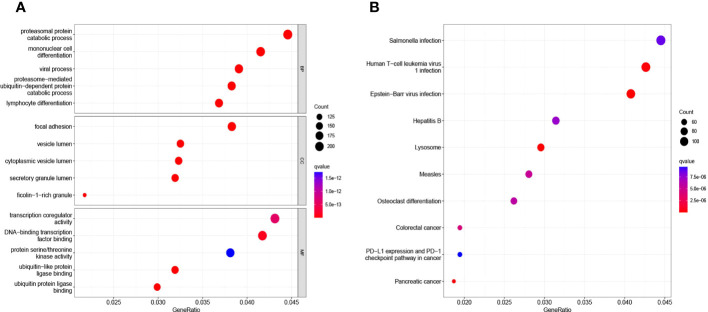
GO analysis **(A)** and KEGG analysis **(B)** of 50 DEGs via the ClusterProfile.

We used the ClusterProfile R software package to perform GO analysis and KEGG analysis on the 50 DEGs. The results showed that the DEGS were mainly involved in proteasomal protein catabolism process, proteasomal protein catabolic process, viral process, proteasome−mediated ubiquitin−dependent protein catabolic process, lymphocyte differentiation, focal adhesion, vesicle lumen, vesicle lumen, secretory granule lumen, transcription coregulator activity, transcription coregulator activity (**Figure 2A**). In addition, KEGG analysis showed some signaling pathways, including the Salmonella infection pathway, Human T−cell leukemia virus 1 infection, Epstein−Barr virus infection, were also significantly different between the two cohorts (**Figure 2B**).

### 3.3 Determination and verification of diagnosis markers

**Figure 3:**
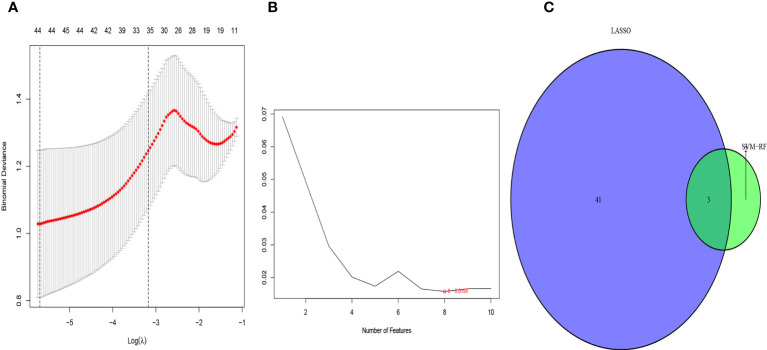
Selection of diagnosis marker candidates for sepsis: **(A)** tuning feature screening in the LASSO model; **(B)** a plot of biological marker screening via the SVM-RFE arithmetic; **(C)** Venn graph displaying 3 diagnosis biomarkers shared by LASSO and SVM-RFE.

We used the LASSO regression algorithm to interrogate the 50 DEGs for diagnostic markers of sepsis, which uncovered 44 potentially diagnostically relevant genes (**Figure 3A**). We also analyzed the 50 DEGs using the SVM-RFE algorithm, which identified eight feature subsets (**Figure 3B**). Only three genes were identified by both of the approaches: COMMD9, CSF3R, and NUB1 (**Figure 3C**), suggesting these genes may be involved in sepsis diagnose.

### 3.4 The expression and diagnosis significance of COMMD9, CSF3R, NUB1 in sepsis

**Figure 4:**
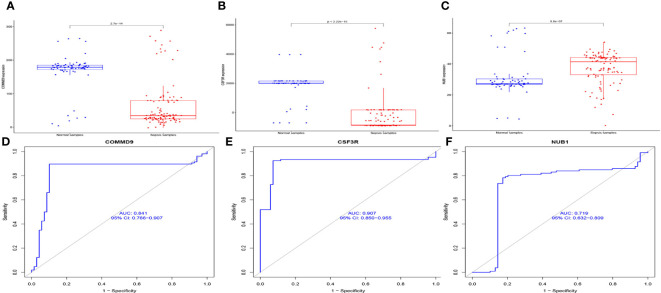
The expression and diagnosis significance of COMMD9、CSF3R、NUB1 in Sepsis: **(A)** COMMD9 expression was distinctly downregulated in Sepsis samples; **(B)** CSF3R expression was distinctly downregulated in Sepsis samples; **(C)** NUB1 expression was distinctly upregulated in Sepsis samples; **(D–F)** ROC assays for COMMD9, CSF3R, NUB1 in Sepsis.

We found that, compared with normal samples, the expression levels of COMMD9 and CSF3R were all significantly down-regulated in samples from patients with sepsis compared to control samples (**Figures 4A, B**). NUB1 was significantly up-regulated in the samples (**Figure 4C**). We next performed ROC analysis of COMMD9, CSF3R, NUB1, and the results showed that COMMD9 (**Figure 4D**, AUC=0.841), CSF3R (**Figure 4E**, AUC=0.907), NUB1 (**Figure 4F**, AUC=0.719).

### 3.5 COMMD9, CSF3R, NUB1 are related to immunocyte infiltration levels

**Figure 5:**
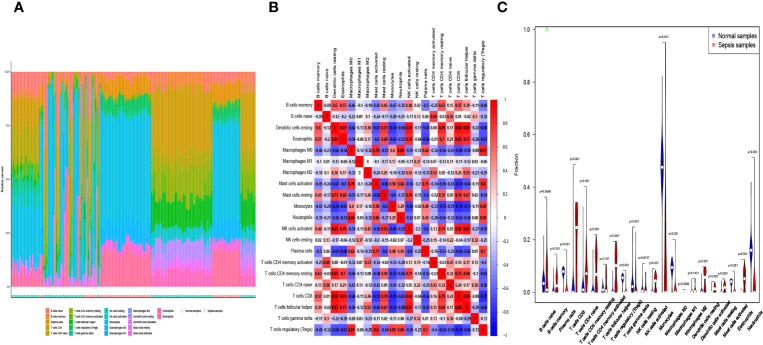
**(A, B)** The percentage of the 22 immunocytes identified via the CIBERSORT arithmetic. **(C)** The diversities in the architecture of immunocytes between Normal and Sepsis samples.

**Figure 6:**
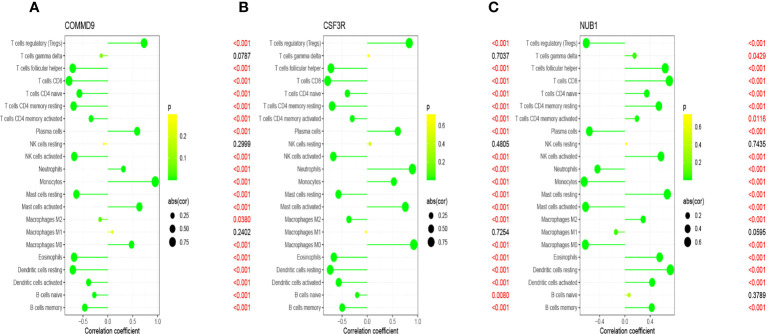
Correlation between key genes and infiltrating immune cells in sepsis and normal samples. **(A)** COMMD9 and infiltrating immune cells; **(B)** CSF3R and infiltrating immune cells; **(C)** NUB1 and infiltrating immune cells.s

Infiltration of immunocytes in the tissue microenvironment is an independent prognostic indicator of overall survival. We therefore investigated the relationship between COMMD9, CSF3R, and NUB1 expression and the infiltration of immune cells in specimens from patients with sepsis and healthy controls. CIBERSORT was used to characterize the immune cell composition of the two cohorts (**Figures 5A, B**). And we also found some statistically significant differences in B cells memory, Plasma cells, T cells CD8, T cells CD4 native, T cells CD4 memory resting, T cells follicular helper, T cells regulatory, T cells gamma delta, NK cells activated, Monocytes, Macrophages M0, Dendritic cells resting, Dendritic cells activated, Mast cells resting, Mast cells activated, Eosinophils, Neutrophils (**Figure 5C**).This demonstrated that there is a correlation between lower expression of COMMD9, CSF3R and NUB1 and increased immune cell infiltration (**Figures 6A–C**). These data suggest that COMMD9, CSF3R, and NUB1 may regulate immune cells in patients with sepsis.

### 3.6 The identification of the expression of three diagnostic genes in our cohort

**Figure 7:**
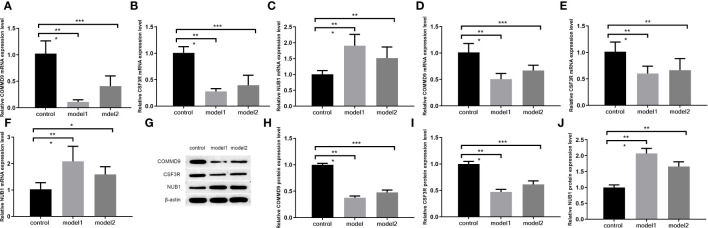
The qRT-PCR **(A–F)** and WB **(G–J)** for the levels of key genes. **(A–C)** The mRNA level of COMMD9, CSF3R, NUB1 in ileum tissue; **(D–F)**_The mRNA level of COMMD9 , CSF3R, NUB1 in serum; **(G–J)** The protein level of COMMD9、CSF3R、NUB1 in ileum tissue (*p<0.05、**p<0.01、***p<0.001).

The PCR results in rat ileum tissue show that,compared with control group samples, the expression levels of COMMD9 and CSF3R were significantly decreased from rats with two sepsis model (LPS group=model1, CLP group=model2) (**Figures 7A, B**), and the expression level of NUB1 was significantly increased (**Figure 7C**).

The PCR results in rat serum show that, compared with control group samples, the expression levels of COMMD9 and CSF3R were significantly decreased from rats with two sepsis model (LPS group=model1, CLP group=model2) (**Figures 7D, E**), and the expression level of NUB1 was significantly increased (**Figure 7F**).

The WB results show in rat ileum tissue that (**Figure 7G**), compared with control group samples, the expression levels of COMMD9 and CSF3R were significantly decreased from rats with two sepsis model (LPS group=model1, CLP group=model2)(**Figures 7H, I**), and the expression level of NUB1 was significantly increased (**Figure 7J**).

## 4 Discussion

Sepsis is a systemic inflammatory response syndrome caused by bacteria and other pathogenic microorganisms infiltrating the blood. It often occurs secondary to other critical illness and severe infection of organs or tissues. There are currently no reliable biomarkers for early detection and diagnosis of sepsis and, because sepsis can quickly lead to death, there is an urgent need for new approaches to identify the disease as early as possible. To this end, in this study, we evaluated datasets from four microarrays of patients with sepsis and healthy subjects to identify DEGs that may have diagnostic value. GO analysis results show that 50 DEGS mainly involve in the processing of proteomic protein catabolic, proteasomal protein catabolic process, viral process, proteasome−mediated ubiquitin−dependent protein catabolic process, lymphocyte differentiation, focal adhesion, vesicle lumen, vesicle lumen, secretory granule lumen, transcription coregulator activity, transcription coregulator activity. And KEGG analysis showed some signaling pathways, including the Salmonella infection pathway, Human T−cell leukemia virus 1 infection, Epstein−Barr virus infection. We uncovered 50 DEGs between the cohorts that we further analyzed, resulting in prioritization of COMMD9, CSF3R, and NUB1 as potential biomarkers to diagnose sepsis.

This study is the first to propose and verify differential expression of COMMD9, CSF3R, and NUB1 in patients with sepsis, and ROC analysis confirmed that they may be useful as diagnostic biomarkers. The Copper Metabolism Murr1 Domain (COMMD) protein family plays an important role in the regulation of nuclear factor-κB (nf-κB) activity, copper ion metabolism, and sodium ion problems, and mutations in this gene have been associated with related biological phenotypes (11–13). The nf-κB signaling pathway is one of the most important signaling pathways in sepsis. Therefore, we speculate that COMMD9 plays a role in the nf-κB signaling pathway, and we will investigate whether it is related in the future. Colony-Stimulating Factor-3 Receptor (CSF3R) is a protein-coding gene located in the p34 region of chromosome 1 that regulates the generation, differentiation, and signal transduction of granulocytes (14). At present, CSF3R research is mainly focused on its role in blood diseases, especially in the blood of patients with chronic neutrophilic leukemia (15, 16). CSF3R is also involved in cancer progression (17). However, the expression and role of CSF3R in patients with sepsis are not known. NEDD8 Ultimate Buster-1 (NUB1) is an interferon-inducible gene whose overexpression inhibits cell growth. It is known to play an important role in cancer and cell physiology (18–20), but its role in sepsis is not clear.

Sepsis primarily manifests as a severe inflammatory response. In addition to inflammatory cells, a large number of B cells, T cells and other immune cells are observed in the tissue infiltrate, and they each play different roles. Indeed, immune cell infiltration is one of the critical mechanisms that induces sepsis (21, 22). CD4+ T cells are thought to be the most prone to cell death during sepsis, thereby inducing an immune paralysis state (23, 24).After activation of CD4+ T cells in the intestinal mucosa, T regulatory cells decrease, which in turn hyperactivates the immune response, eventually damaging the intestinal epithelial cells (25). Studies have found that recurrent sepsis can deplete human CD4+ T cells, thereby changing the prognosis of patients (26). Other immune cells have different pathogenic or protective effects. Therefore, identifying new immunotherapeutic targets by characterizing immune cell infiltration is a priority. In this study, we found that COMMD9, CSF3R, and NUB1 regulate immune cell infiltrate during sepsis, and we speculate that the activity of these genes may influence the development of sepsis. Future pre-clinical and clinical studies are needed to verify their effectiveness and interrelationships.

We recognize some limitations of this study. First, our study was based on a bioinformatics analysis of microarray data made available by others and validated with only one small animal study. Larger-scale experiments and mechanistic evaluation are needed to validate the potential role of DEGs in sepsis. Second, the four microarrays varied from one another with regard to patient country, race, and gender. Third, the correlation we identified between three DEGs and immune cell infiltrate is reported here as a phenomenon, and rigorous studies to determine how those genes influence immune cell behavior in the context of sepsis—and whether this differs from their role in a non-pathological context—are required.Due to insufficient funding, we were unable to verify the relationship between key genes and immune cells.

We identified COMMD9, CSF3R, and NUB1 as potentially biologically relevant genes and diagnostic markers for sepsis. Our findings prompt additional studies of these three genes to understand how they may contribute to the pathological mechanisms of sepsis onset and progression and to identify novel opportunities to treat sepsis by targeting aberrant immune cell behaviors.

## Data availability statement

The datasets presented in this study can be found in online repositories. The names of the repository/repositories and accession number(s) can be found below: https://www.ncbi.nlm.nih.gov/, GSE1175, https://www.ncbi.nlm.nih.gov/, GSE28750, https://www.ncbi.nlm.nih.gov/, GSE48080, https://www.ncbi.nlm.nih.gov/, GSE12624.

## Ethics statement

The animal study was reviewed and approved by Ethics Committee of Hebei University of Traditional Chinese Medicine (Number: DWL2019023).

## Author contributions

QW and CW: manuscript preparation, data analysis, and the research conception. JM, FC: manuscript revision. YL, ZL, DL: conception and design. WZ, YT, JG: data analysis. All authors contributed to the article and approved the submitted version.
